# Wheat endosperm-specific transcription factor TaDOF6 enhances grain development by regulating *TaSWEET13h* expression and facilitating sugar and gibberellin transport

**DOI:** 10.3389/fpls.2025.1608090

**Published:** 2025-06-25

**Authors:** Run Ding, Tongtong Xiao, ShaSha Li, Jian Qiang, Heng Zhang, Hongmiao Chang, Yueming Yan, Xiaohui Li

**Affiliations:** College of Life Science, Capital Normal University, Beijing, China

**Keywords:** wheat, seed size, DOF transcription factor, SWEET transport protein, transcriptional regulation, molecular dynamics simulations

## Abstract

Seed size is regulated by the coordinated growth of the seed coat, embryo, and endosperm, and is modulated by multiple factors. Plant hormones, sugars, and cell cycle-related processes play key roles in this regulation. In this study, we demonstrate that overexpressing the endosperm-specific DOF transcription factor gene *TaDOF6* significantly enhances the accumulation of sugars and gibberellin (GA_3_) in grains during the grain-filling stage. RNA sequencing (RNA-seq), quantitative real-time PCR (RT-qPCR), yeast one-hybrid (Y1H), electrophoresis mobility shift assay (EMSA), and dual-luciferase assays further confirmed that *TaSWEET13h* is a direct downstream target of TaDOF6. Structural and functional analyses identified TaSWEET13h as a multifunctional cell membrane-localized transporter that transports diverse soluble sugars and GA_3_. Notably, molecular dynamics (MD) simulations and *in vitro* assays revealed that hydrophobic interactions among non-polar amino acids primarily drive the transport of sucrose and GA_3._ Therefore, these findings elucidate the genetic regulatory network involving SWEET sugar transporters in grain size control and highlight promising targets for high-yield wheat breeding.

## 1 Introduction

Wheat, domesticated from wild ancestors during the Neolithic era, has been cultivated for over 10,000 years (Shewry, 2009). Its remarkable adaptability enables widespread global cultivation (Levy and Feldman, 2022). Wheat flour serves as a primary ingredient in numerous staple foods—including bread, buns, noodles, pancakes, cakes, and cookies—making it a key dietary energy source (Veraverbeke and Delcour, 2002; Shewry and Hey, 2015). Therefore, elucidating the mechanisms of seed development and identifying genes that enhance quality and yield are essential for breeding programs aimed at increasing wheat production and mitigating food shortages.

DOF (DNA-binding with one finger) proteins constitute a plant-specific class of transcription factors (TFs) (Yanagisawa, 1995). Genome-wide analyses have revealed the presence of DOF family members across multiple plant species, including mouse-ear cress (*Arabidopsis thaliana*) (Yanagisawa, 2002), rice (*Oryza sativa*) (Yanagisawa, 2002), sorghum (*Sorghum bicolor*) (Kushwaha et al., 2011), birch (*Betula platyphylla*) (Sun et al., 2021), watermelon (*Citrullus lanatus*) (Zhou et al., 2020), foxtail millet (*Setaria italica*) (Zhang et al., 2017), spinach (*Spinacia oleracea*) (Yu et al., 2021), and rose (*Rosa chinensis*) (Nan et al., 2021). Recently, Liu et al. (2020) identified 96 *DOF* genes in wheat through whole-genome analysis and classified them into five subfamilies based on phylogenetic and functional characteristics. *DOF* genes participate in diverse plant processes, including stress responses (Zang et al., 2017), seed development (Bueso et al., 2016), germination (Boccaccini et al., 2014; Gardiner et al., 2010), hormone signaling (Song et al., 2016), light responses (Yanagisawa and Sheen, 1998), and metabolic regulation (Tanaka et al., 2009), thereby playing a critical role in plant growth and development.

Chen et al. (2010) identified a novel sugar transporter in *Arabidopsis thaliana* using a glucose-based fluorescence resonance energy transfer (FRET) sensor, naming it SWEET (Sugars Will Eventually be Exported Transporter). SWEET proteins facilitate sugar transport across membranes along concentration gradients between intracellular and extracellular compartments, independent of proton gradients (Bermejo et al., 2011; Chen et al., 2012). Consequently, their activity does not depend on environmental pH. These transporters also mediate bidirectional sugar flux driven by solute potential gradients (Bermejo et al., 2011; Chen et al., 2012). In contrast, other sugar transporters, including MSTs and SUTs, require proton coupling and function unidirectionally along proton concentration gradients between cellular compartments (Kühn and Grof, 2010; Ayre, 2011; Slewinski, 2011).

SWEET proteins are conserved across prokaryotes, animals, and plants, though gene numbers vary significantly. Prokaryotes and animals typically harbor few SWEET genes. For example, *Mycoplasma arthritidis*, *Prochlorococcus marinus*, *Mus musculus*, *Papio anubis*, and humans each possess only one (Yuan and Wang, 2013; Patil, 2015), while *Drosophila melanogaster* has two and *Caenorhabditis elegans* has seven (Chen et al., 2010). In contrast, higher plants contain many *SWEET* genes, including 17 in *Arabidopsis* (Chen et al., 2010), 21 in rice (Yuan and Wang, 2013), 24 in maize (Liu et al., 2022; Zhu et al., 2022), and 108 in wheat (Gautam et al., 2019).

Multiple studies have demonstrated that DOF TFs are key regulators of grain size. In crops such as maize, rice, and sorghum, DOF TFs influence endosperm development and nutrient metabolism by modulating starch synthase-related genes (Zhang et al., 2016; Qi et al., 2017; Wu et al., 2019). In rice, tissue-specific overexpression of *OsDOF11* activates *SWEET14*, enhancing both yield and disease resistance (Kim et al., 2021). Furthermore, Moehs et al. (2019) applied the non-transgenic Targeting Induced Local Lesions in Genomes (TILLING) method to generate a wheat *WPBF* triple-deficient mutant, which exhibited significantly reduced grain size, thousand-grain weight, and starch content compared to the wild type. However, the molecular mechanisms by which DOF TFs regulate endosperm and embryo size remain unclear.

Seed size and shape are essential for both plant reproduction and dispersal, as well as for key agronomic traits. In wheat, grain size positively correlates with grain weight. Seed size is regulated by the coordinated growth of maternal tissues, the embryo, and the endosperm, and is modulated by several factors (Li et al., 2019). Among these, plant hormones, sugars, and cell cycle-related processes play major roles (Zhao et al., 2022; Wang et al., 2020; Xu et al., 2019; Ren et al., 2019).

Our previous work showed that endosperm-specific overexpression of the wheat *TaDOF6* gene enhances grain width, thousand grain weight, and starch content (Ding et al., 2024). Building on this, this study explores the role of *TaDOF6* in grain development and its underlying molecular mechanisms. *TaDOF6* overexpression alters the expression of sugar transporter-related genes and elevates soluble sugar and hormone levels. Notably, TaDOF6 binds to the *TaSWEET13h* promoter, regulating its expression during seed filling. Furthermore, we identified TaSWEET13h as a transporter of both soluble sugars and gibberellins (GA_3_), and this study provides a detailed analysis of its substrate transport mechanism.

## 2 Materials and methods

### 2.1 Plant materials and growth conditions

Wheat: Chinese Spring (CS) was used for gene amplification, Fielder was utilized for protoplast preparation and expression pattern analysis, and a transgenic wheat line overexpressing *TaDOF6* specifically in the endosperm under the *1Dx5* promoter has been previously constructed (Ding et al., 2024). Tobacco: *Nicotiana benthamiana* was employed for transient expression assays. *Arabidopsis*: Columbia (*Col-0*) was used for target gene amplification. Wheat plants were cultivated at the transgenic plant pilot base of the Beijing Agro-Biotechnology Research Center. *Arabidopsis* and tobacco were grown in the Capital Normal University greenhouse under controlled conditions: 24°C/18°C with a 16 h light/8 h dark photoperiod and 50–60% relative humidity.

### 2.2 Measurement of physiological and biochemical indicators

For metabolite extraction, 4 g of 15 days post-anthesis (DPA) wheat grains were placed in a pre-chilled mortar at –20°C and ground to a fine powder in liquid nitrogen. Subsequently, 10 mL of pre-chilled 80% methanol (Sigma-Aldrich, USA) was added, and the mixture was extracted at 4°C for 18 h. After centrifugation (6,000 rpm, 4°C, 10 min), the supernatant was collected into a fresh 50 mL tube. The precipitate was re-extracted with 8 mL of pre-chilled 80% methanol at 4°C for 10 min, followed by a second centrifugation under the same conditions. The resulting supernatants were pooled. A C18 column (Anavo, China) was activated with 4 mL of acetonitrile (Sigma-Aldrich, USA), then washed with 4 mL of distilled water. The combined supernatant was loaded onto the column, which was subsequently washed with 1 mL of distilled water and eluted with 2 mL of 45% methanol. The eluate was filtered through a 0.45 µm membrane (Millipore, USA) and used for subsequent analyses. Gibberellin A3 (GA_3_), auxins, and cytokinins were quantified using ELISA kits (Biotopped, China).

### 2.3 RNA-seq

Total RNA was extracted from 15 DPA grains of transgenic (*TaDOF6* ^OE2^) and Fielder lines using three biological replicates. cDNA synthesis, library construction, sequencing, and primary data analysis were conducted by Novogene Co., Ltd. (Beijing, China). Clean reads were aligned to the CS reference genome. Differentially expressed genes (DEGs) were identified using the R package DEGseq, with a fold change ≥ 2 and adjusted *p*-value (padj) < 10^-3^. RNA-seq data are available at NCBI under accession number PRJNA1248066.

### 2.4 RNA extraction and RT-qPCR

Total RNA was extracted using the Plant RNA Extraction Kit (TaKaRa Bio Inc., Otsu, Shiga, Japan). First-strand cDNA was synthesized with TaKaRa PrimeScript™ RT Master Mix. RT-qPCR was performed on a BioRad CFX96 real-time system (Bio-Rad Laboratories, Inc., Hercules, CA, USA) using SYBR Green qPCR Master Mix (TransGen Biotech, Beijing, China). The thermal cycling conditions included an initial denaturation at 95 °C for 5 min, followed by 40 cycles of 15 s at 95°C and 30 s at 60°C. The relative gene expression was calculated using the 2^-ΔΔCt^ method, with the *Ubiquitin* gene as the internal control. Primers used for RT-qPCR are listed in **Supplementary Table 1**.

### 2.5 Yeast one-hybrid assay

The coding sequence of *TaDOF6* was cloned into the *Sma* I (TaKaRa) enzyme-linearized pGADT7-Rec vector using homologous recombination. The promoter region of *TaSWEET13h* was inserted between the *EcoR* I and *Spe* I sites of the pHis2.1 vector to drive *LacZ* reporter expression. The primers used for PCR amplification are listed in **Supplementary Table 2**. The recombinant plasmid was transformed into yeast strain Y187, which was cultured on SD/-Trp/-Leu solid medium at 30°C for three days. Three randomly selected single colonies were then grown in SD/-Trp/-Leu liquid medium to an OD of 0.6–0.8. Subsequently, 5 μL of the culture were spotted onto SD/-Trp/-Leu/-His solid medium supplemented with 30 mM 3-Amino-1,2,4-triazole (3-AT) and incubated upside down at 30 °C for three days.

### 2.6 Protein induction and purification

The *TaDOF6* coding sequence was also inserted into the *Xho* I site of the pETMALc-H vector. The recombinant protein was expressed in *E. coli* BL21 (DE3) cells (TransGen Biotech). Following cell lysis, His-tagged fusion proteins in the supernatant were captured using Anti-His magnetic beads (Beyotime Biotechnology, Wuhan, China), followed by washing and elution. Protein purification was conducted according to the previously optimized protocol (Zhu et al., 2023).

### 2.7 Western blot

The method described by Zhu et al. (2023) was followed with minor modifications. The 6×His-TaDOF6-MBP protein samples, expressed in *E. coli* BL21 (DE3) and purified as described above, were mixed with 6× loading buffer (Beyotime), separated by 12% SDS-PAGE, and transferred onto a PVDF membrane using the wet Trans-Blot Turbo Transfer System (Bio-Rad). The membrane was blocked with 5% (w/v) skim milk in TBST (20 mM Tris-HCl, pH 7.6, 150 mM NaCl, 0.05% Tween-20) at room temperature for 1 h, then incubated overnight at 4°C with an Anti-6×His primary antibody (Abcam, Cambridge, UK). After three washes with TBST, the membrane was incubated for 1 h at room temperature with the appropriate horseradish peroxidase (HRP)-conjugated secondary antibody (TransGen Biotech). Following another three TBST washes, protein bands were visualized using the Pierce™ ECL Plus Western Blotting Substrate (Thermo Fisher).

### 2.8 Electrophoretic mobility shift assays

Biotin-labeled, cold, and mutant probes were synthesized by Shanghai Sangong Bioengineering Co., Ltd. Probes were diluted to 100 μM in nuclease-free water. Forward and reverse strand probes were then mixed at a 1:1 ratio and annealed in a PCR machine (Bio-Rad Laboratories, Inc., Hercules, CA, USA) by heating to 95°C for 5 min, followed by cooling at 0.1°C every 8 s to 25°C, then stored at 4°C. The probes were purified using the DNA Probe Purification Kit (Omega Bio-tek, Inc., Norcross, GA, USA). Finally, gel electrophoresis was performed as described previously (Zhang et al., 2016).

### 2.9 Dual-luciferase reporter gene assay

Genomic DNA (gDNA) was extracted from wheat leaves using the CTAB method (Zhang et al., 2013). A 1,000 bp promoter region upstream of the *TaSWEET13h* start codon (*pTaSWEET13h*) was amplified and cloned into the *Kpn* I and *Xho* I sites of the pGreenII-0800-LUC vector. The resulting *pTaSWEET13h::LUC* construct served as the reporter, with the *Renilla luciferase* (*REN*) gene under the *35S* promoter included as an internal control. The *TaDOF6* coding sequence (CDS) was inserted into the *Xba* I and *Kpn* I sites of the pGreenII-62SK vector to generate the *35S::TaDOF6* effector. Dual-luciferase assays were performed in *Nicotiana benthamiana* leaves following Yang et al. (2025). *Agrobacterium tumefaciens* cells carrying the constructs were resuspended in infiltration buffer (10 mM MgCl_2_, 10 mM MES, 150 μM acetosyringone, ddH_2_O) to an OD_600_ of ~0.5. After 3 days, luciferase (LUC) and REN activities were measured using the Dual-Luciferase^®^ Reporter Assay System (Promega, Madison, WI, USA) on a multifunctional microplate reader (Thermo Fisher Scientific, Waltham, MA, USA). The LUC/REN ratio was calculated using the empty vector (62SK) plus *pTaSWEET13h* as the control.

### 2.10 Transmembrane structure prediction

The SWEET transporter protein sequence was submitted to TMHMM (https://services.healthtech.dtu.dk/services/TMHMM-2.0/) for transmembrane domain prediction. Additionally, AlphaFold 2 (Jumper et al., 2021) was used to model the three-dimensional (3D) structure of TaSWEET13h.

### 2.11 Subcellular localization

The TaDOF6 CDS was cloned into the *Bam*H I-digested 16318h-*GFP* vector (TaKaRa), provided by Dr. Yimiao Tang (Beijing Academy of Agriculture and Forestry Sciences). The resulting 16318h-*TaDOF6*-*GFP* and empty 16318h-*GFP* vectors were introduced into wheat leaf protoplasts using PEG4000, as described by Zhu et al. (2023). After 16–18 h of dark incubation at 25°C, GFP and mCherry signals were detected using an FV1000MPE confocal laser scanning microscope (Olympus, Tokyo, Japan).

### 2.12 Molecular docking

AlphaFold 2 was employed to model TaSWEET13h as the receptor protein for molecular docking. Subsequently, molecular docking of small molecule ligands—sucrose, glucose, fructose, galactose, mannose, and GA_3_—retrieved from https://pubchem.ncbi.nlm.nih.gov/ was conducted using AutoDockTools-1.5.7 and AutoDock Vina. The receptor protein was protonated and assigned partial charges in AutoDock, while the ligands were similarly hydrogenated and charged at the root. The prepared receptor was then docked with the ligands. Top-ranking binding conformations, based on AutoDock Vina scoring, were selected for further analysis. Finally, the resulting docked complexes were visualized in cartoon mode using PyMol (Schrödinger, Inc., New York, NY, USA).

### 2.13 Yeast compensation experiment

Sucrose and hexose transport assays were carried out using sugar transporter-deficient yeast mutant strains *Sey6210* and *EBY.VW4000*, provided by Dr. Xueli An (University of Science and Technology Beijing) and Dr. Yong Xu (Beijing Academy of Agriculture and Forestry Sciences), respectively. These strains were transformed with pDR196 (negative control), pDR196-*AtSWEET13* (positive control; gift from Dr. Xueli An), or pDR196-*TaSWEET13h* using the standard LiAc/PEG method. Three independent colonies were used per assay. For *Sey6210*, colonies were grown overnight at 30°C in SD/-Ura liquid medium, serially diluted to 10^4^, 10^3^, 10^2^, and 10 cells/µL, and 5 µL aliquots were spotted onto SC/-Ura plates containing 2% glucose and 2% sucrose. For *EBY.VW4000*, colonies were cultured overnight at 30°C in SC/-Ura medium supplemented with 2% (v/v) maltose. The cell suspensions with four concentration gradients were spotted onto SC/-Ura plates containing 2% maltose, 2% glucose, 2% fructose, 2% galactose, or 2% mannose. Plates were incubated at 30°C for three days and then photographed.

GA_3_ transport assays were conducted using a modified yeast three-hybrid (Y3H) system as described previously (Kanno et al., 2016). The yeast strain Y2HGold (Weidi Biotechnology Co., Ltd., Shanghai, China) was co-transformed with pGADT7-*AtGAI* and pGBKT-*AtGID1a*, representing components of the GA signaling pathway, along with pDR196 (negative control) or pDR196-*TaSWEET13h*. Colonies were cultured overnight at 30°C in SD/-Trp/-Leu liquid media, serially diluted to 10^4^, 10^3^, 10^2^, and 10 cells/µL, and 5 µL of each dilution from three independent colonies was spotted onto SD/-Trp/-Leu/-His/-Ade and SD/-Trp/-Leu/-His/-Ade/+GA_3_ plates containing 30 mM 3-AT.

### 2.14 Esculin uptake assay

The sucrose transport activity of TaSWEET13h was assessed using the fluorescent sucrose analog esculin, following the method of Huai et al. (2022). Yeast mutants (*Sey6210*) transformed with pDR196-*TaSWEET13h* and control mutants carrying the empty vector pDR196 were incubated in an esculin buffer (1 mM esculin in 25 mM sodium phosphate buffer, pH 4.0) for 1 h. After washing, cells were imaged using a confocal fluorescence microscope with 405 nm excitation and 488 nm emission.

### 2.15 Molecular dynamics simulation

MD simulations of the TaSWEET13h–sucrose/GA_3_ complex were performed using GROMACS 2022.3 (GROningen MAchine for Chemical Simulations, Department of Biochemistry, University of Groningen) on the high-performance computing platform at the Inner Mongolia High Performance Computing Public Service Platform (Huhehot, China) to obtain equilibrated conformations for subsequent MM/PBSA calculations. Trajectory analysis and image generation were conducted using PyMol (Schrödinger, Inc., New York, NY, USA) and OriginPro (OriginLab Corporation, Northampton, MA, USA).

Binding free energy and per-residue energy decomposition of the TaSWEET13h-sucrose/GA_3_ complex were calculated using the gmx_mmpbsa module based on the MM/PBSA method. The binding free energy (Δ*G_bind_*) was calculated using the formula: Δ*G_bind_* = Δ*E_MM_* + Δ*G_PB_* + Δ*G_SA_* - *T*Δ*S*, where Δ*E_MM_* represents the electrostatic and van der Waals energy in vacuum, while Δ*G_PB_* and Δ*G_SA_* denote the differences in polar and nonpolar solvent solvation free energies, respectively. The entropic contribution (-*T*Δ*S*) was excluded due to its computational cost and minimal variation in identical protein systems. To identify key binding residues, per-residue free energy contributions were decomposed into van der Waals and electrostatic energies (Δ*G_vdw_* and Δ*G_ele_*), polar solvation free energy (Δ*G_PB_*), and nonpolar solvation free energy (Δ*G_SA_*). Residues contributing more than 1 kcal·mol^-1^ to Δ*G_bind_* were considered critical for binding affinity.

### 2.16 Amino acid directed mutagenesis

To generate the pDR196-*TaSWEET13hmu* plasmid, the TaSWEET13h CDS (from 16318h-*TaSWEET13h*) was site-specifically mutated at active amino acid residues using the KOD-Plus Mutagenesis Kit (TOYOBO, product code SMK-101).

## 3 Results

### 3.1 Overexpression of *TaDOF6* promotes the soluble sugar accumulation and the expression of genes encoding sugar transporters

**Figure 1:**
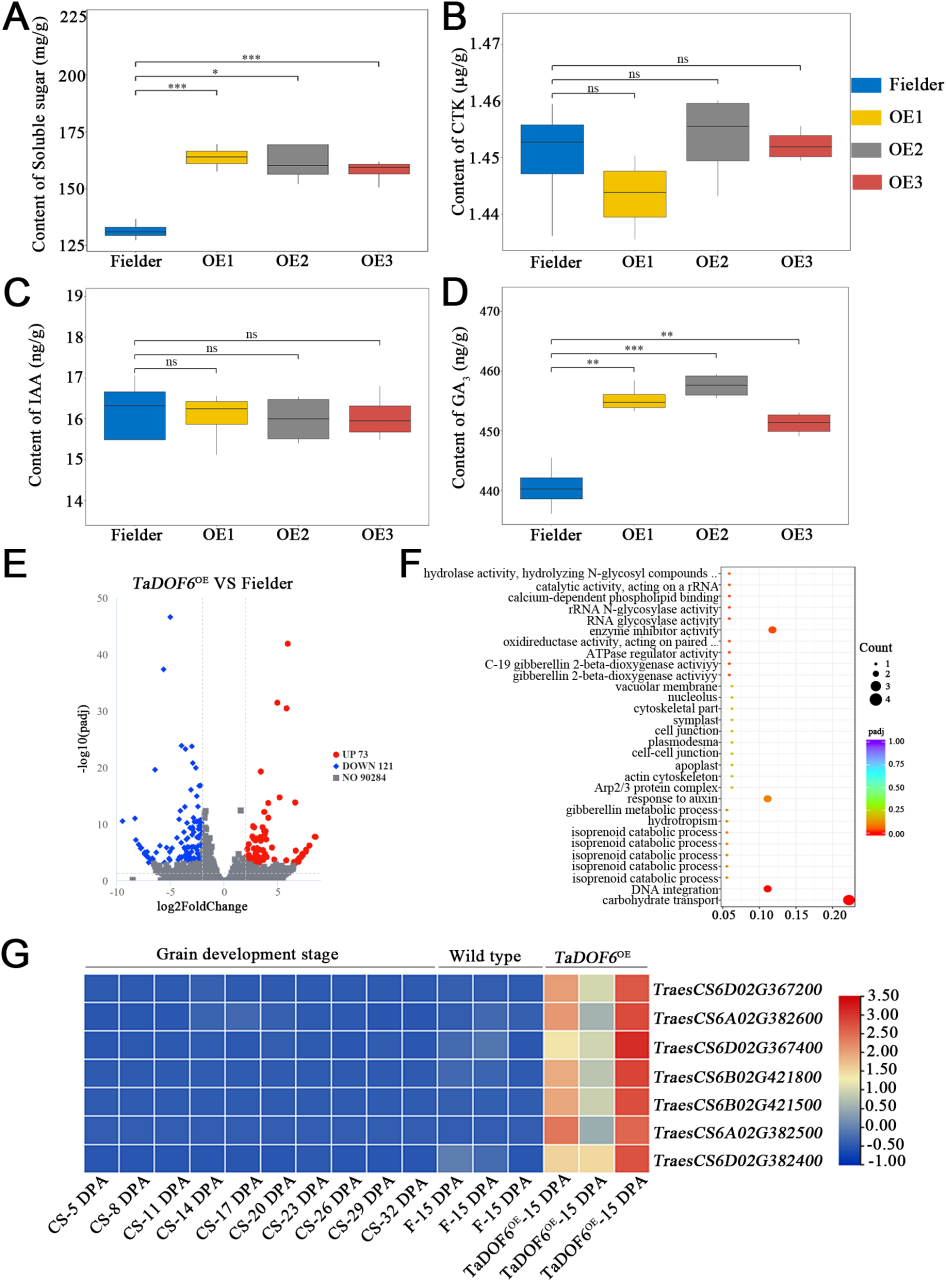
Soluble sugar and hormone accumulation and RNA-seq analysis of *TaDOF6* transgenic lines and Fielder. **(A–D)** soluble sugar content, cytokinin content, auxin content, and GA_3_ content, respectively; data are mean ± Standard deviation (SD), *n* = 6; **(E)** Volcano plot analysis of DEGs; **(F)** GO annotation analysis of upregulated genes in transgenic lines; **(G)** Expression patterns of sugar transport-related genes in 15 DPA grains of transgenic lines and Fielder, and the development endosperm of CS wheat (PRJNA545291). Asterisks indicate significant differences using the Student’s *t*-test (*p < 0.05; **p < 0.01; ***p < 0.001).

To investigate the role of TaDOF6 in wheat grain development, we analyzed soluble sugar content and endogenous hormone levels in grains of *TaDOF6* overexpression transgenic lines during the grain filling stage. Overexpression of *TaDOF6* significantly increased soluble sugar and GA_3_ levels in 15 DPA grains, while auxin and cytokinin levels remained unchanged (**Figures 1A–D**). To further elucidate the biological function of TaDOF6, we performed RNA-seq analysis on 15 DPA grains from overexpression lines and their transformation receptor, Fielder. The GC contents were 51.21–54.17% (**Supplementary Table S3**). Pearson’s correlation analysis was conducted to assess inter-sample relationships (**Supplementary Figure S1**). In total, 194 differentially expressed genes (DEGs) were identified, including 73 up-regulated and 121 down-regulated DEGs (**Figure 1E**). Gene Ontology (GO) annotation of upregulated DEGs revealed enrichment in pathways associated with carbohydrate transport, enzyme inhibitor activity, auxin response, and other biological processes (**Figure 1F**). Notably, all genes involved in the carbohydrate transport pathway belonged to the SWEET gene family. We further examined the transcriptional profiles of these *SWEET* genes in developing endosperm of Chinese Spring (CS) (PRJNA545291) (Gu et al., 2021) (**Figure 1G**). The results showed that TaSWEET13h expression was generally low in grains during the filling stage of wild-type wheat, but relatively high in vegetative tissues such as roots, stems, and leaves. Importantly, *TaSWEET13h* expression was significantly upregulated in grains of TaDOF6 overexpression lines during the filling stage, in stark contrast to wild type (**Figure 1G**, **Supplementary Figures S2**, **S3**), indicating that *TaDOF6* overexpression significantly increased markedly enhances *TaSWEET* genes expression in the endosperm.

### 3.2 TaDOF6 activates *TaSWEET13h* expression by binding to the P-box motif in wheat

**Figure 2:**
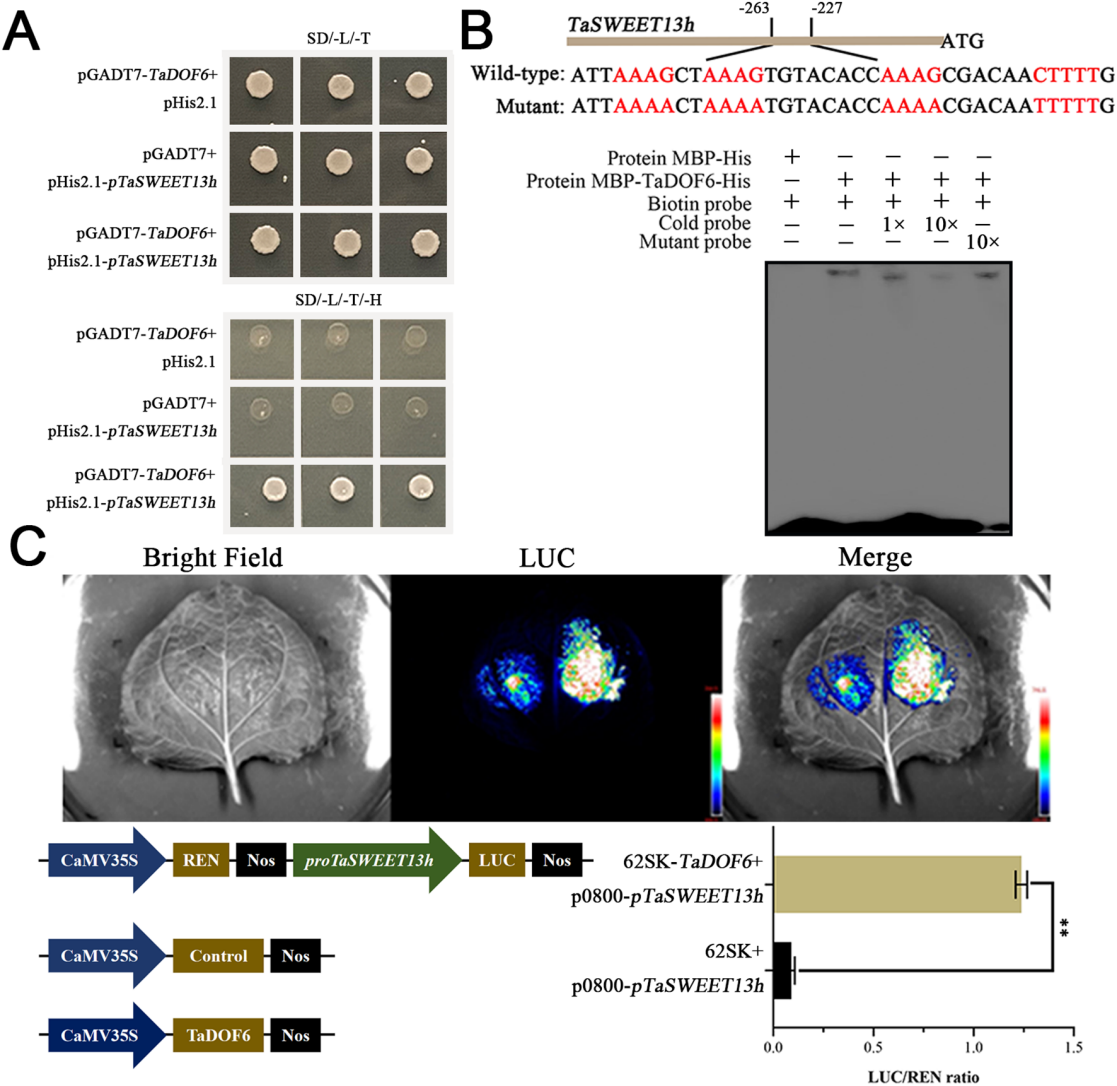
Analysis of TaDOF6 binding to the *TaSWEET13h* promoter. **(A)** Yeast one hybrid assay assessing the binding of TaDOF6 to the *TaSWEET13h* promoter. Interaction was tested on SD medium lacking leucine, tryptophan and histidine, with pGADT7-*TaDOF6* plus pHis2.1, and pGADT7 plus pHis2.1-*pTaSWEET13h* used as negative controls. **(B)** EMSA demonstrating the specific binding of TaDOF6 to the P-box motif within the *TaSWEET13h* promoter. The red-highlighted part in the sequence is the P-box. **(C)** Dual-luciferase reporter assay showing the activation effect of TaDOF6 on the *TaSWEET13h* promoter. The LUC/REN activity of pGreenII-62SK plus *35S::REN*-*pTaSWEET13h*::LUC was used as a negative control; data are mean ± SD, *n* = 6. Asterisks indicate significant differences using the Student’s *t*-test (**p < 0.01).

Y1H assay was conducted to determine whether the transcription factor TaDOF6 binds to the promoter region of TaSWEET13h. The coding sequence of *TaDOF6* was fused with GAL4AD, while the *TaSWEET13h* promoter was fused with the *His* reporter gene. In three independent experiments, transformants co-expressing pGADT7-*TaDOF6* and pHis2.1-*pTaSWEET13h* survived on SD/-Trp/-Leu/-His medium supplemented with 3-AT, demonstrating that *TaDOF6* directly binds to the *TaSWEET13h* promoter (**Figure 2A**). This binding was further confirmed by EMSA, which showed interaction between TaDOF6 and the “AAAG” motif in the *TaSWEET13h* promoter (**Supplementary Figure S4**, **Figure 2B**). In *Nicotiana benthamiana* leaves, co-transformation with pGreenII-62SK-*TaDOF6* and pGreenII-0800-*pTaSWEET13h* significantly increased the LUC/REN ratio by 833.33% compared to the negative control (**Figure 2C**). Together, these results confirm that TaDOF6 directly binds to the *TaSWEET13h* promoter and regulates its expression.

### 3.3 TaSWEET13h is cell membrane-localized

**Figure 3:**
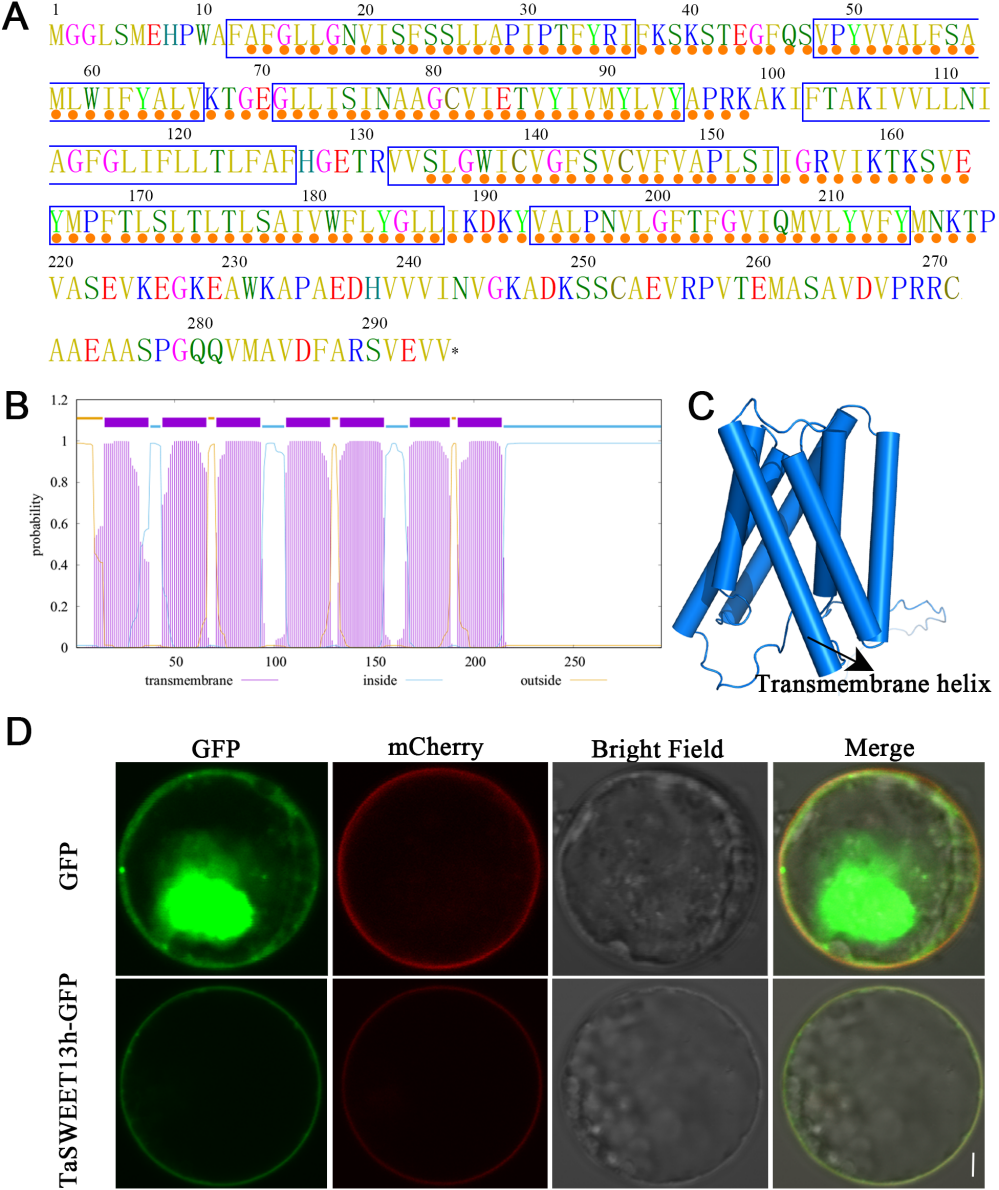
Structure and localization analysis of TaSWEET13h transporter. **(A)** TaSWEET13h protein sequence. Blue boxes and orange dots represent the transmembrane domain and MtN3_slv domains, respectively. **(B)** TMHMM analysis of the transmembrane domain profile of TaSWEET13h. **(C)** Cartoon image of the 3D structure of TaSWEET13h resolved by AlphaFold 2. **(D)** Subcellular localization of TaSWEET13h in wheat leaf protoplasts. The protoplasts transfected with the 16318h-*GFP* plasmid are used as a control, with mCherry fluorescence representing the marker of cytoplasmic membrane. Scale bar=5 μm.

InterProScan analysis identified two MtN3_slv domains within the TaSWEET13h protein (13–98 aa and 134–218 aa), characteristic of the plant SWEET family (**Figure 3A**), indicating that TaSWEET13h is a SWEET family member. Predictions from TMHMM and AlphaFold 2 showed that TaSWEET13h contains seven transmembrane domains (located at 12–36 aa, 48–66 aa, 72–94 aa, 106–126 aa, 132–153 aa, 165–187 aa, and 193–214 aa), with the N- and C-termini positioned on opposite sides of the membrane (**Figures 3A–C**). Upon transformation of the 16318h-*TaSWEET13h-GFP* construct into wheat leaf protoplasts, GFP fluorescence localized to the cytoplasmic membrane, whereas the negative control 16318h-*GFP* showed diffuse fluorescence throughout the cell (**Figure 3D**). These findings confirm that TaSWEET13h is a cell membrane -localized protein.

### 3.4 Molecular structure of TaSWEET13h for substrate transport

**Figure 4:**
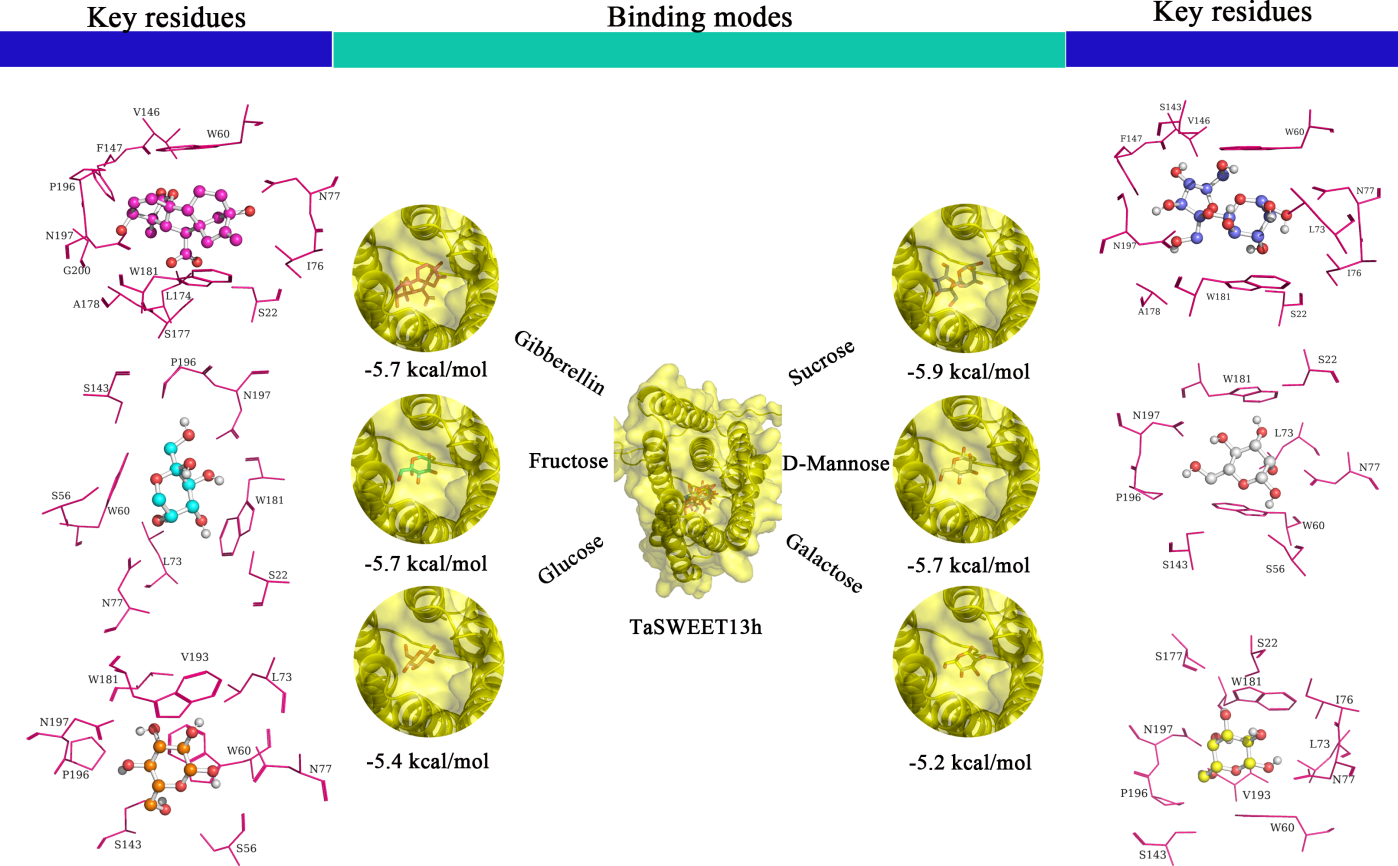
Binding modes and key residues of TaSWEET13h to different substrates. Substrates include GA_3_ (red), sucrose (purple), fructose (blue), mannose (white), glucose (orange), and galactose (yellow). Amino acid residues within 4 Å of the substrates are highlighted in magenta.

To investigate the role of TaSWEET13h in small molecule transport, we used its AlphaFold 2-predicted 3D structure as the receptor for molecular docking. Using AutoDock Vina, we calculated the binding energies of various ligands, including sucrose, glucose, fructose, galactose, mannose, and GA_3_. All ligands exhibited negative binding energies and hydrogen bond distances below 4 Å (**Figure 4**), indicating strong interactions with TaSWEET13h. These results suggest that different substrates may share the same transport channel on TaSWEET13h, potentially leading to competitive inhibition when multiple substrates are present.

### 3.5 TaSWEET13h has a substrate transport function

**Figure 5:**
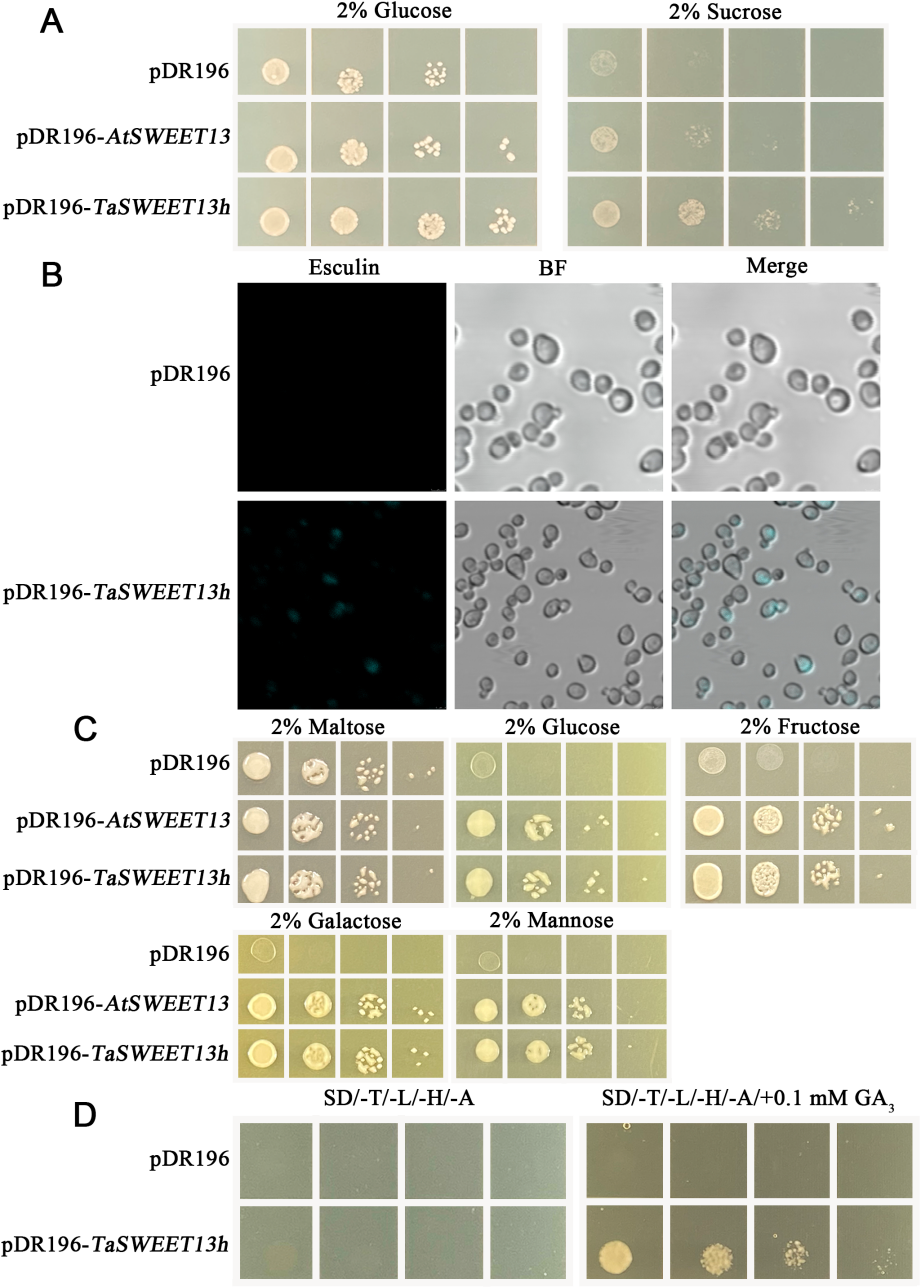
Soluble sugars and GA_3_ transport assays of TaSWEET13h in *Saccharomyces cerevisiae*. **(A)** Growth assays of yeast strains transformed with pDR196-*TaSWEET13h*, pDR196-*AtSWEET13h* (positive control), or pDR196 (negative control) on SC/-Ura plate containing 2% glucose and 2% sucrose. **(B)** Uptake assay of esculin by yeast containing the pDR196-*TaSWEET13h* plasmid or pDR196 empty vector. Yeast cells were incubated in 25 mM sodium phosphate buffer (pH 4.0) containing 1 mM esculin for 1 h. Cells was observed under the confocal microscopy after washing. Scale bar=5 μm. **(C)** Growth assays of yeast strains transformed with pDR196-*TaSWEET13h*, pDR196-*AtSWEET13h* (positive control), or pDR196 (negative control) on the SC/-Ura plate containing 2% maltose, glucose, fructose, or mannose. **(D)** Growth assays of yeast co-transformed with pGADT7*-AtGAI* plus pGBKT*-AtGID1a* and either pDR196-*TaSWEET13h* or pDR196 on the SD/-Trp/-Leu/-His/-Ade plate containing 0.1 mM GA_3_. 10× represents the number of dilutions of the bacterial solution. Yeast cultures were serially diluted and spotted onto plates, starting with an initial OD_600_ of 0.2, followed by 10-fold serial dilutions for each subsequent spot.

The yeast mutant strain *Sey6210*, which lacks the extracellular invertase gene *SUC2*, cannot hydrolyze sucrose and thus fails to grow on media where sucrose is the sole carbon source (Milne et al., 2013). All transformants harboring pDR196 (empty vector), pDR196-*AtSWEET13*, or pDR196-*TaSWEET13h* grew well on glucose-supplemented media. However, when glucose was replaced with sucrose, *Sey6210* strains expressing pDR196-*TaSWEET13h* exhibited significantly higher growth rates than those carrying the empty vector (**Figure 5A**). To further verify whether *TaSWEET13h* transports sucrose, we performed an esculin uptake assay in *Sey6210*. Under confocal fluorescence microscopy, fluorescence accumulated inside TaSWEET13h-expressing cells, whereas no fluorescence was detected in the negative control (**Figure 5B**), confirming that TaSWEET13h facilitates sucrose transport.

Similarly, the yeast mutant strain EBY.VW4000, which lacks all hexose transporter genes, cannot grow on media containing hexoses as the sole carbon source. While all transformants, including those carrying pDR196, pDR196-*AtSWEET13*, or pDR196-*TaSWEET13h*, grew comparably on maltose-containing media, growth differed on media containing glucose, fructose, galactose, or mannose. The *EBY.VW4000* strains transformed with the pDR196 empty vector had significantly lower growth than those transformed with pDR196-*TaSWEET13h* (**Figure 5C**).

GID1 (gibberellin insensitive dwarf 1) and DELLA proteins are central components of the GA signaling pathway in plants, with their interaction forming its core regulatory mechanism. Under low endogenous GA levels, DELLA proteins accumulate and repress the expression of growth-related genes, thereby inhibiting plant growth. Conversely, elevated GA levels allow GA to bind its receptor, activating the GID protein complex. This complex interacts with DELLA proteins, promoting their degradation and relieving repression on growth-related genes, which facilitates plant growth and development. To investigate this interaction, we co-transformed Y2HGold yeast strains with the pDR196 empty vector or pDR196-*TaSWEET13h*, along with pGADT7-*AtGAI* and pGBKT-*AtGID1a*. Strains carrying the pDR196 empty vector or pDR196-*TaSWEET13h* failed to grow on SD/-Trp/-Leu/-His/-Ade medium without GA_3_. However, those transformed with pDR196-*TaSWEET13h* grew on SD/-Trp/-Leu/-His/-Ade medium supplemented with 0.1 mM GA_3_ (**Figure 5D**). These results indicate that TaSWEET13h is capable of transporting sucrose, glucose, fructose, galactose, mannose, and GA_3_.

### 3.6 Stability and dynamics of TaSWEET13h-sucrose/GA_3_ complexes in MD simulation

**Figure 6:**
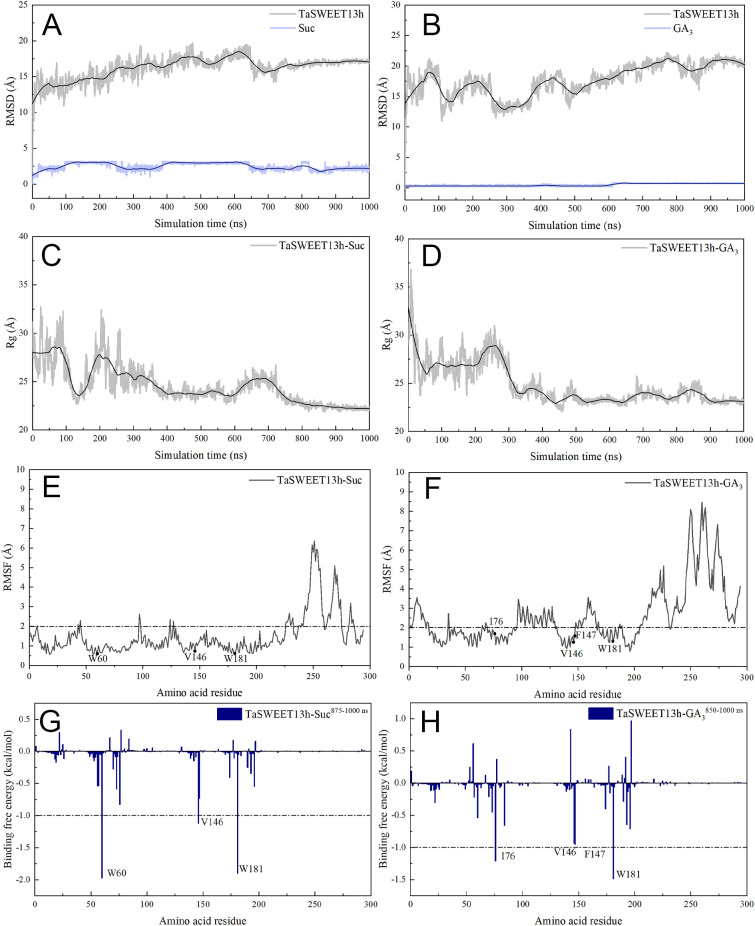
MD simulations of the TaSWEET13h-sucrose/GA_3_ complexes. **(A, B)** The time-evolution RMSD curves of TaSWEET13h-sucrose/GA_3_ complexes during CHARMM36m MD simulations. **(C, D)** The Rg curves of TaSWEET13h-sucrose/GA_3_ complexes during CHARMM36m MD simulations. **(E, F)** The RMSF curves of TaSWEET13h-sucrose/GA_3_ complexes during CHARMM36m MD simulations. The residue contributions exceeding -1 kcal.mol^-1^ to the binding free energy are marked. **(G, H)** Total binding free energy (Δ*G_bind_*) contributions of TaSWEET13h-sucrose/GA_3_ complexes. Each residue for the TaSWEET13h-sucrose/GA_3_ complexes calculated from the equilibrated conformations during independent MD run with CHARMM36m force fields. The residue contributions exceeding -1 kcal.mol^-1^ to the binding free energy are marked.

To investigate the transport mechanisms and key residues of the TaSWEET13h protein for sucrose and GA_3_, we conducted a 1000 ns MD simulation using the optimal binding conformations of TaSWEET13h docked with sucrose and GA_3_ as initial structures. Analysis of the simulation trajectories revealed that the root mean square deviation (RMSD) and radius gyration (Rg) revealed that the RMSD of the TaSWEET13h-Suc and TaSWEET13h–GA_3_ complexes stabilized at 875 ns and 850 ns, respectively, with average RMSD values of 17 Å and 21 Å. The standard errors of these values remained below 1 Å post-equilibration (**Figures 6A, B**). Similarly, the Rg reached equilibrium at 750 ns and 870 ns, with respective averages of 22 Å and 23 Å, and standard errors also under 1 Å (**Figures 6C, D**). These results indicate that both complexes attained stable conformations by the end of the 1000 ns of MD simulation. To further characterize their dynamic behavior, we performed conformation sampling and clustering analysis (**Supplementary Figure S5**). Five representative conformations of sucrose and GA_3_ were tightly embedded within the hydrophobic cavity of TaSWEET13h’s active site. Minor fluctuations in ligand positioning were driven by directional shifts in the hydrophobic side chains of active site residues.

We calculated the binding free energy (Δ*G_bind_*) of the TaSWEET13h-Suc and TaSWEET13h-GA_3_ complexes, which comprises four components: van der Waals energy (Δ*G_vdw_*), electrostatic Coulomb energy (Δ*G_ele_*), solvation free energy (Δ*G_PB_*), and non-polar solvation free energy (Δ*G_SA_*). This analysis reveals the contributions of these energy components to the binding process of TaSWEET13h with sucrose and GA_3_. In both complexes, Δ*G_vdw_* was significantly higher (TaSWEET13h-Suc: -16.54 kcal·mol^-1^; TaSWEET13h-GA_3_: -16.09 kcal·mol^-1^) than the other components. Δ*G_ele_* and Δ*G_PB_* were weaker, with values for TaSWEET13h-Suc of -7.78 kcal·mol^-1^ and -1.51 kcal·mol^-1^, respectively, and for TaSWEET13h-GA_3_ of -2.28 kcal·mol^-1^ and -1.75 kcal·mol^-1^, respectively. The Δ*G_SA_* values for TaSWEET13h-Suc and TaSWEET13h-GA_3_ were +14.68 kcal·mol^-1^ and +12.72 kcal·mol^-1^, respectively, suggesting that polar residues adversely affect binding (**Supplementary Table S4**). These results highlight the crucial role of van der Waals interactions in the binding process of the TaSWEET13h-Suc/GA_3_ complexes.

To identify the amino acid residues in TaSWEET13h essential for binding sucrose and GA_3_, the MM/PBSA method was employed to decompose the binding free energy into residue-specific contributions within the TaSWEET13h-Suc and TaSWEET13h-GA_3_ complexes. As shown in **Figure 6E**, W60, V146, and W181 contributed significantly to binding in TaSWEET13h–Suc, with Δ*G_bind_* values of 1.97, -1.12, and -1.90 kcal·mol^-1^, respectively. In the TaSWEET13h-GA_3_ complex, four amino acid residues (I76, V146, F147, and W181) showed higher contributions, with Δ*G_bind_* values of -1.22, -1.94, -0.96, and -1.49 kcal·mol^-1^, respectively (**Figure 6F**). Consistent with overall binding free energy, hydrophobic interactions of each residue were critical in both complexes, particularly for W60 and W181 (Δ*G_MM_* < -2.00 kcal·mol^-1^). Notably, the indole ring of W181 engaged in both hydrophobic interactions and hydrogen bonding with sucrose and GA_3_, enhancing complex stability. Other residues did not contribute to the binding affinity to sucrose and GA_3_. However, S56 and G84 displayed distinct roles in the two complexes. For sucrose and GA₃, Δ*G_bind_* of S56 were -0.54384 and 0.61992 kcal·mol^-1^, respectively, while those of G84 were +0.19152 and -0.6636 kcal·mol^-1^, respectively. During the 1000 ns MD simulation of the TaSWEET13h-Suc/GA_3_ complexes, the root mean square fluctuation (RMSF) values of residues W60, I76, V146, F147, and W181 were all < 2.0 Å; whereas the RMSF values of residues outside the active site were generally higher (**Figures 6G, H**). Therefore, this further indicated that these key residues interact with sucrose and GA_3_ to form stable and reasonable complexes.

### 3.7 Verify affinity activity sites of TaSWEET13h to substrates by the yeast expression system

**Figure 7:**
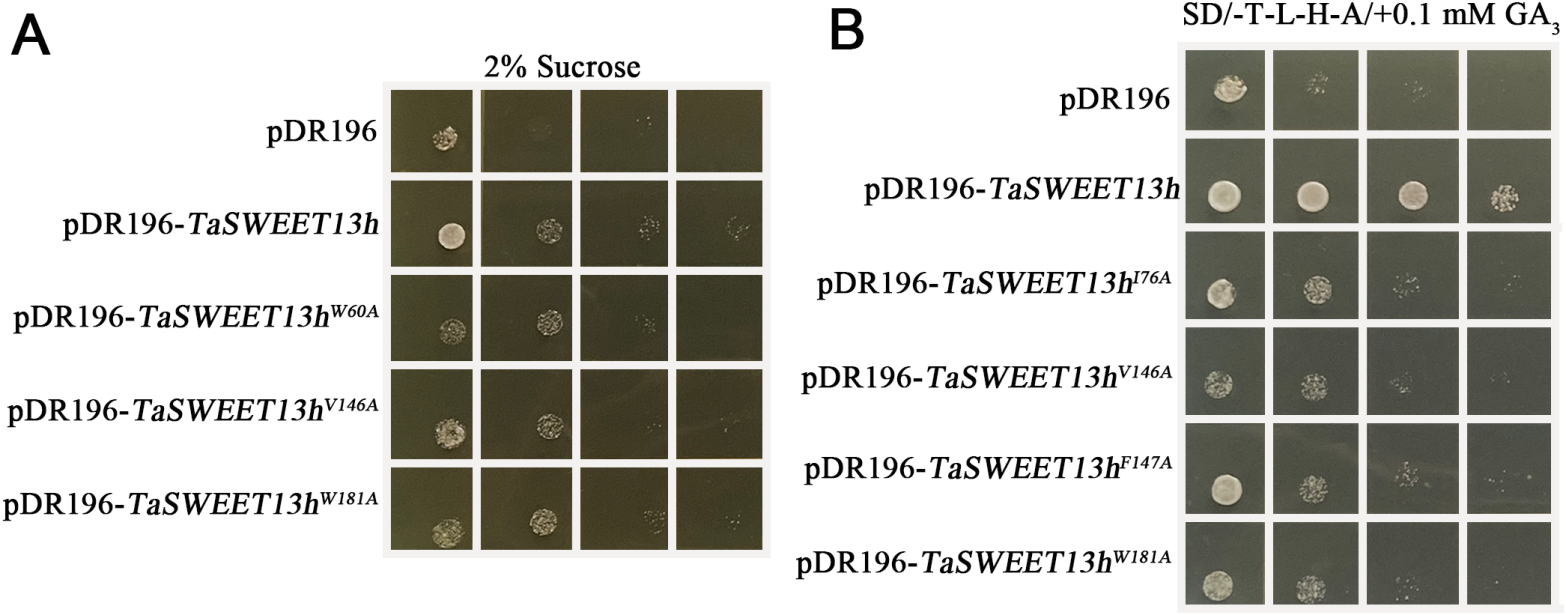
Screening substrate binding sites of TaSWEET13. **(A)** Serial dilution assays were performed with *Sey6210* yeasts carrying either pDR196-*TaSWEET13mu* (W60A, V146A, or W181A) or pDR196-*TaSWEET13h*. Yeast cells were grown for 3 d on SC/-Ura medium containing 2% sucrose. **(B)** Serial dilution assays were performed with *Y2HGold* yeasts carrying pGBKT7-*AtGID1a*, pGADT7-*AtGAI*, and either pDR196-*TaSWEET13mu* (I76A, V146A, F147A, or W181A) or pDR196-*TaSWEET13h*. Yeast cells were grown for 3 d on SD/-Leu/-Trp/-His/-Ade medium containing 0.1 µM GA_3_. Yeast cultures were serially diluted and spotted onto plates, starting with an initial OD_600_ of 0.2, followed by 10-fold serial dilutions for each subsequent spot.

Based on MD simulation results, three residues (W60, V146, and W181) and four residues (I76, V146, F147, and W181) were mutated to assess the affinity of TaSWEET13h for sucrose and GA_3_, respectively. Compared to *Sey6210* [pDR196-*TaSWEET13h*], strains expressing mutated TaSWEET13h [pDR196-*TaSWEET13hmu*] exhibited reduced growth on selection medium with sucrose as the sole carbon source (**Figure 7A**). Similarly, compared to Y2HGold [pGADT7-*AtGAI*; pGBKT-*AtGID1a*; pDR196-*TaSWEET13h*], the mutant strains [pGADT7-*GAI*; pGBKT-*GID1a*; pDR196-*TaSWEET13hmu*] showed reduced growth on medium containing 0.1 mM GA_3_ (**Figure 7B**). These results indicate that the V146 and W181 residues are critical for TaSWEET13h’s affinity to sucrose and GA_3_.

## 4 Discussion

### 4.1 TaDOF6 promotes *TaSWEET13h* expression by binding to the P-box motif

Grain weight is a key agronomic trait influencing crop yield and is controlled by conserved molecular pathways (Long et al., 2024). Elucidating these pathways will enhance the regulatory network of yield traits and support molecular breeding for high yield (Zhang et al., 2024). As grain size strongly correlates with grain weight, it has remained a central focus in genetic and breeding research. Our previous studies identified *TaDOF6* as a DOF family TF that is specifically and highly expressed in wheat grains, regulating carbohydrate accumulation, grain size, and weight (Ding et al., 2024), although its exact regulatory mechanisms remain unclear. In rice, OsDOF11 directly regulates the sucrose transport genes *OsSUT1*, *OsSWEET11*, and *OsSWEET14*, thereby modulating sucrose transport (Wu et al., 2018). OsDOF11 also plays a role in seed development, and its tissue-specific overexpression increases grain weight by activating *SWEET14* expression (Kim et al., 2021). This study investigates the positive regulatory role of *TaDOF6* during grain filling. RNA-seq and RT-qPCR analyses confirmed that *TaDOF6* overexpression significantly elevates TaSWEET13h transcript levels. Further Y1H, EMSA, and dual-luciferase assays demonstrated that TaDOF6 binds to the P-box motif. Thus, we propose that TaDOF6 enhances *TaSWEET13h* transcription by binding the P-box motif in the developing wheat endosperm.

### 4.2 TaSWEET13h is involved in multiple substrate transport

SWEET family members, characterized by seven transmembrane helices and sugar transport activity, play a critical role in grain development. In maize and rice, SWEET4 transports hexoses across the endosperm cell membrane during grain filling, promoting larger grain size (Sosso et al., 2015). In *Arabidopsis*, AtSWEET11, AtSWEET12, and AtSWEET15 are expressed in the seed coat and endosperm to import sucrose into the embryo, supporting normal seed development. Triple mutants of these genes exhibit delayed embryo development and reduced seed weight, starch, and lipid contents (Chen et al., 2015). In soybean, overexpression of dominant alleles *GmSWEET10a*, *GmSWEET10b*, and *GmSWEET39* increases seed size and oil content (Miao et al., 2020; Wang et al., 2019). Similarly, OsSWEET11, 14, and 15 contribute to carbohydrate transport into rice grains; knockout mutants of *ossweet11*, *14*, and *15* show abnormal grain filling and reduced seed weight and starch content (Ma et al., 2017; Yang et al., 2018; Fei et al., 2021; Li et al., 2022). In lychee, spatiotemporal expression profiling suggests that *LcSWEET2a* and *LcSWEET3b* participate in seed development. In wheat, knockout of *TaSWEET11* downregulates genes involved in starch biosynthesis and sucrose metabolism, resulting in impaired starch accumulation, pericarp shrinkage, and significantly reduced sucrose levels in *tasweet11-ko* lines (Wang et al., 2025). Collectively, these findings suggest that wheat SWEET proteins are key, functionally conserved sucrose transporters that facilitate efficient sucrose translocation during grain filling, thereby supporting grain development. Here, we show that TaSWEET13h, a SWEET family member, contains seven transmembrane helices and a conserved *MtN3_slv* domain, consistent with its plasma membrane localization. Previous studies have demonstrated that SWEET proteins transport not only soluble sugars but also hormones. For instance, HvSWEET11 transports both sugars and cytokinins to promote barley grain development (Radchuk et al., 2023), while AtSWEET13 and *OsSWEET3a* facilitate the transport of both sugars and GA (Kanno et al., 2016). In rice, *OsSWEET3a* exhibits dual sugar and GA transport functions, with both knockout and overexpression leading to delayed germination and slow growth (Morii et al., 2020). Consistent with these findings, we report that TaSWEET13h can transport various soluble sugars, including sucrose, glucose, and fructose, as well as gibberellins. These substrates likely bind within a common active pocket of TaSWEET13h, where hydrophobic interactions involving nonpolar amino acid residues serve as the primary driving force for its affinity to sucrose and GA_3_.

**Figure 8:**
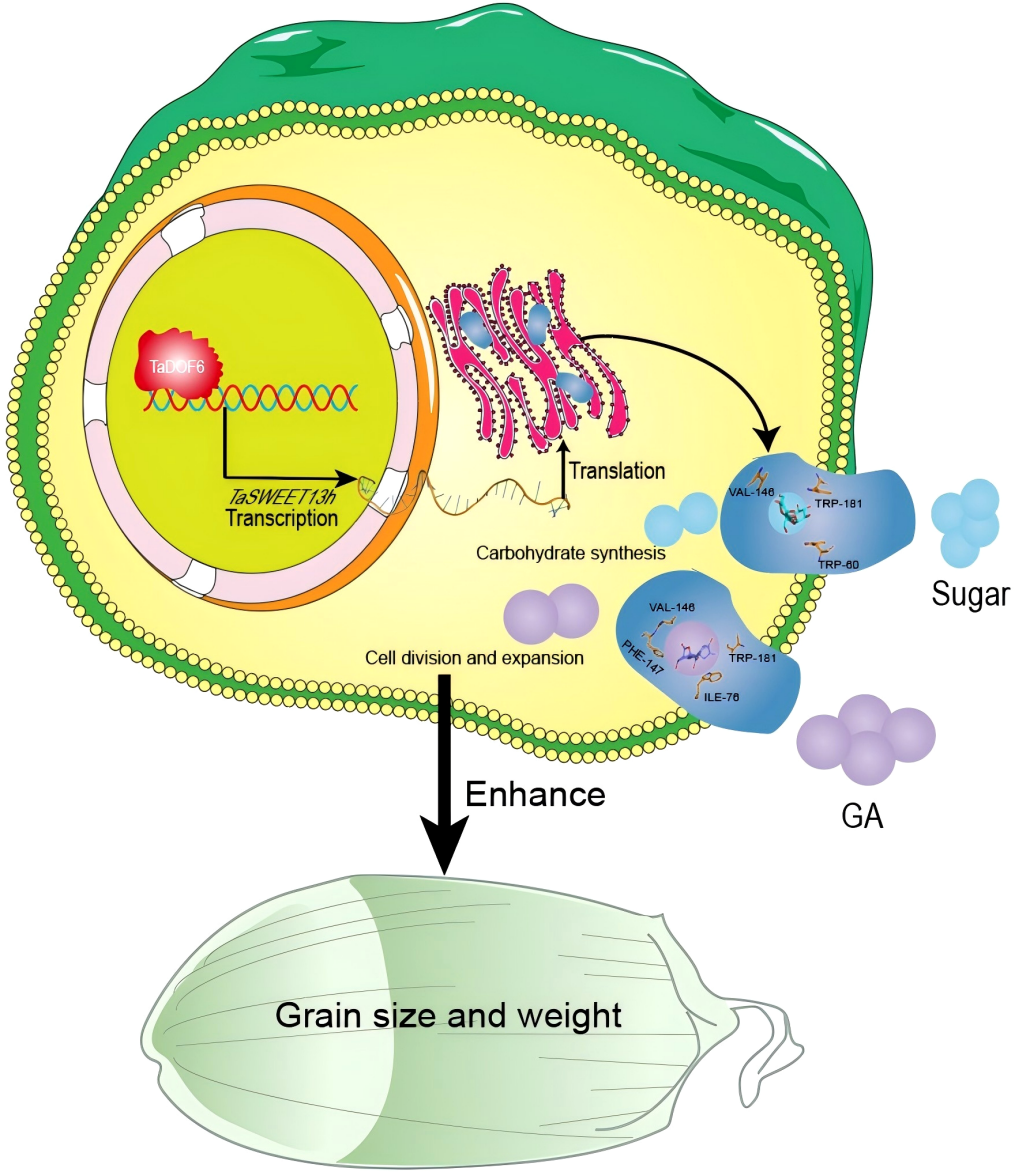
A model diagram of TaDOF6 regulating *TaSWEET3h* expression to promote grain development.

However, due to the current lack of *TaSWEET13h* overexpression and gene-edited lines, its role in regulating wheat grain size cannot be directly validated. Based on existing findings, we propose a hypothetical model illustrating the potential mechanism by which *TaDOF6* and *TaSWEET13h* regulate wheat grain size and weight (**Figure 8**). During grain filling, *TaDOF6* transcript levels increase sharply. TaDOF6 proteins bind to the P-box motif and activate *TaSWEET13h* transcription in the nucleus. The resulting TaSWEET13h protein integrates into the plasma membrane, facilitating the transport of soluble sugars and GA_3_ from the extracellular space into the cytoplasm. High soluble sugar levels support starch accumulation, while gibberellins promote endosperm cell expansion. Together, these coordinated processes regulate wheat grain development. This study advances our understanding of the molecular basis of wheat grain development and offers strategies for improving yield.

## 5 Conclusions

We found that TaDOF6 activates *TaSWEET13h* via the P-box motif and influences the uptake of small molecules such as sugars and GA_3_. Three and four amino acid residues may affect TaSWEET13h’s affinity for soluble sugars and GA_3_, respectively. TaSWEET13h likely uses a shared active-site pocket for transporting diverse small molecules, with hydrophobic interactions among nonpolar residues as the primary driving force. Overall, this study provides theoretical insights for breeding high-yield, high-quality wheat and highlights the potential of DOF transcription factors and SWEET transporters in enhancing carbohydrate accumulation in cereal crops.

## Data Availability

The datasets presented in this study can be found in online repositories. The names of the repository/repositories and accession number(s) can be found in the article/supplementary material.
