# Supplementary material for: Wheat endosperm-specific transcription factor TaDOF6 enhances grain development by regulating TaSWEET13h expression and facilitating sugar and gibberellin transport
Source: Front Plant Sci. 2025 Jun 25;16:1608090. doi: 10.3389/fpls.2025.1608090 (PMC12237909; doi:10.3389/fpls.2025.1608090)
Supplement: Supplementary file 1 [file DataSheet1.docx]

Table S1. The primers designed for RT-qPCR.

| **Primers** | **Forward (5’- 3’)** | **Reverse (5’- 3’)** |
| --- | --- | --- |
| *TaSWEET13h* | GTCAAGGAGGGGAAAGAGGC | AAAGTCCACCGCCATCACTT |
| *Ubiquitin* | TGACACCATCGACAACGTGA | GAGGGTGGACTCCTTCTGGA |

Table S2. The primers designed for PCR amplification.

| **Primers** | **Sequence (5’- 3’)** | **End use** |
| --- | --- | --- |
| *TaSWEET13h*-163-F | TATCTCTAGAGGATCCATGGGTGGCCTATCCATGGA | Subcellular localization |
| *TaSWEET13h*-163-R | TGCTCACCATGGATCCGACCACCTCGACAGAACGG |  |
| pGADT7-*TaDOF6*-F | CAACGCAGAGTGGCCATTATGGCCCATGTTTTGCTGTTGGCCCCA | Y1H |
| pGADT7-*TaDOF6*-R | CGAGGCGGCCGACATGTTTTTTCCCTTACATCAGGGAGCTGCTATTG |  |
| pHis2.1-*TaSWEET13h*-F | GACTCACTATAGGGCGAATTCCTACTCCGCTACTTACAATTTTAGC |  |
| pHis2.1-*TaSWEET13h*-R | ATAATGCCAGGAATTACTAGTAATTGTAAGGTTGGTAATGGGGT |  |
| pETMALc-*TaDOF6*-F | GCTGGTTCCGCGTGGATCCCTCGAGATGTTTTGCTGTTGGCCCCA | Prokaryotic expression |
| pETMALc-*TaDOF6*-R | GTGGTGGTGGTGGTGCTCGAGATCAGGGAGCTGCTATTGA |  |
| Labeled probe-F | Biotin-ATTAAAGCTAAAGTGTACACCAAAGCGACAACTTTTG-Biotin | EMSA |
| Labeled probe-R | Biotin-CAAAAGTTGTCGCTTTGGTGTACACTTTAGCTTTAAT-Biotin |  |
| Mutation probe-F | ATTAAAACTAAAATGTACACCAAAACGACAATTTTTG |  |
| Mutation probe-R | CAAAAATTGTCGTTTTGGTGTACATTTTAGTTTTAAT |  |
| pGreenII-62SK-*TaDOF6*-F | CCGCGGTGGCGGCCGCTCTAGAATGTTTTGCTGTTGGCCCCA | Dual-LUC |
| pGreenII-62SK-*TaDOF6*-R | GATTTCAGCGTACCGAATTGGTACCATCAGGGAGCTGCTATTGA |  |
| PGreenII-0800-LUC-*pTaSWEET13h*-F | TCACTATAGGGCGAATTGGGTACCCTACTCCGCTACTTACAATTTTAGC |  |
| PGreenII-0800-LUC-*pTaSWEET13h*-R | TATCGATACCGTCGACCTCGAGAATTGTAAGGTTGGTAATGGGGT |  |
| W60A-F | CAGTGCGTAGAAGATCGCCAGCATCGCGCTGAAC | Amino acid directed mutagenesis |
| W60A-R | GTTCAGCGCGATGCTGGCGATCTTCTACGCACTG |  |
| I76A-F | AGCCGGCAGCGTTGGCGCTGATGAGGAGGC |  |
| I76A-R | GCCTCCTCATCAGCGCCAACGCTGCCGGCT |  |
| V146A-F | GTGGGGCAACGAAGGCGCAGACGGAGAAG |  |
| V146A-R | CTTCTCCGTCTGCGCCTTCGTTGCCCCAC |  |
| F147A-F | CTGAGTGGGGCAACGGCGACGCAGACGGAGAA |  |
| F147A-R | TTCTCCGTCTGCGTCGCCGTTGCCCCACTCAG |  |
| W181A-F | AGGCCGTAGAGGAACGCGACGATTGCGCTGAG |  |
| W181A-R | CTCAGCGCAATCGTCGCGTTCCTCTACGGCCT |  |

Notes: The underlined bases are the homologous arm sequences.

**Table S3. Quality inspection of transcriptome data**

| Sample | Raw_reads | Clean_reads | Clean_bases (Gb) | Error_rate | Q20 (%) | Q30 (%) | GC_pct (%) |
| --- | --- | --- | --- | --- | --- | --- | --- |
| Fielder1 | 42350180 | 40898950 | 6.13 | 0.03 | 97.24 | 92.78 | 53.16 |
| Fielder2 | 40905870 | 37540346 | 5.63 | 0.03 | 97.08 | 92.43 | 52.01 |
| Fielder3 | 42777648 | 42282912 | 6.34 | 0.03 | 97.30 | 93.02 | 52.21 |
| *TaDOF6*^OE1^ | 44670448 | 41122106 | 6.17 | 0.03 | 96.91 | 92.16 | 51.75 |
| *TaDOF6*^OE2^ | 49636390 | 48007266 | 7.2 | 0.03 | 97.08 | 92.49 | 54.17 |
| *TaDOF6*^OE3^ | 47638374 | 45909136 | 6.89 | 0.03 | 97.52 | 93.26 | 51.21 |

Notes: sample, sample name; raw_reads, the number of reads in the raw data; clean_reads, the number of reads after filtering the raw data; clean_bases, the number of bases after filtering the raw data (clean base = clean reads * 150bp); error_rate, the overall sequencing error rate of the data; Q20, the percentage of bases with a Phred value greater than 20 in the total number of bases; Q30, the percentage of bases with a Phred value greater than 30 in the total number of bases; GC_pct, the percentage of G and C among the four bases in clean reads.

| **Complex** | **Cluster (ns)** | **Van der waal energy (ΔG*_vdw_*)** | **Ele energy (ΔG*_ele_*)** | **Polar solvation energy (ΔG*_PB_*)** | **SASA energy (ΔG*_SA_*)** | **Binding energy (ΔG*_bind_*)** |
| --- | --- | --- | --- | --- | --- | --- |
| **TaSWEET13h-Suc** | 875-1000 | -16.54 | -7.78 | 14.68 | -1.51 | -11.15 |
| **TaSWEET13h-GA_3_** | 850-1000 | -16.09 | -2.28 | 12.72 | -1.75 | -7.41 |

Table S4. Binding free energy of TaSWEET13h-Sucrose/GA_3_ complex

Notes: The unit of all energies is kcal·mol^-1^.


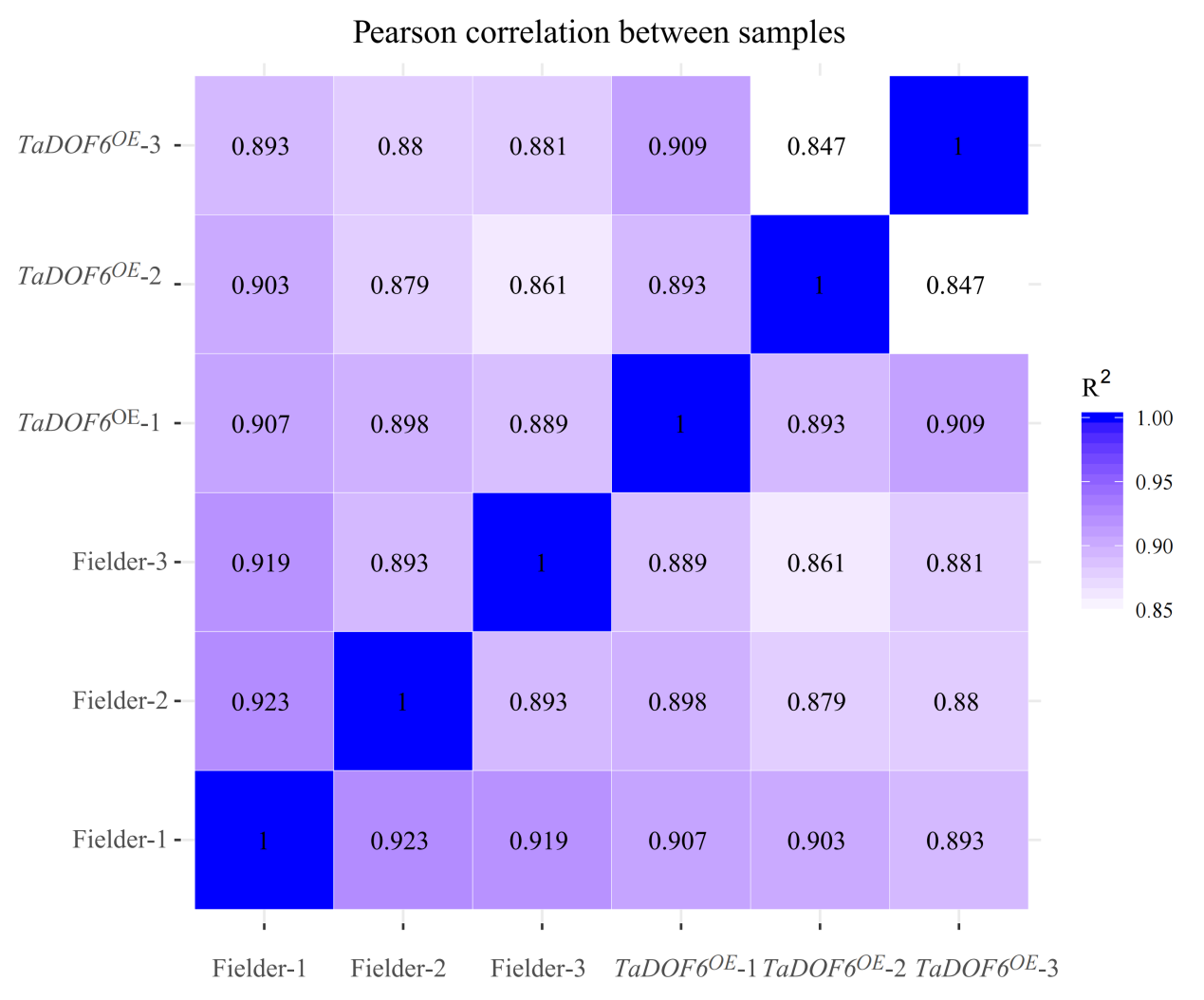


Fig. S1. Transcriptome sample correlation analysis diagram. Pearson correlation coefficients were used to evaluate the similarity between two samples.


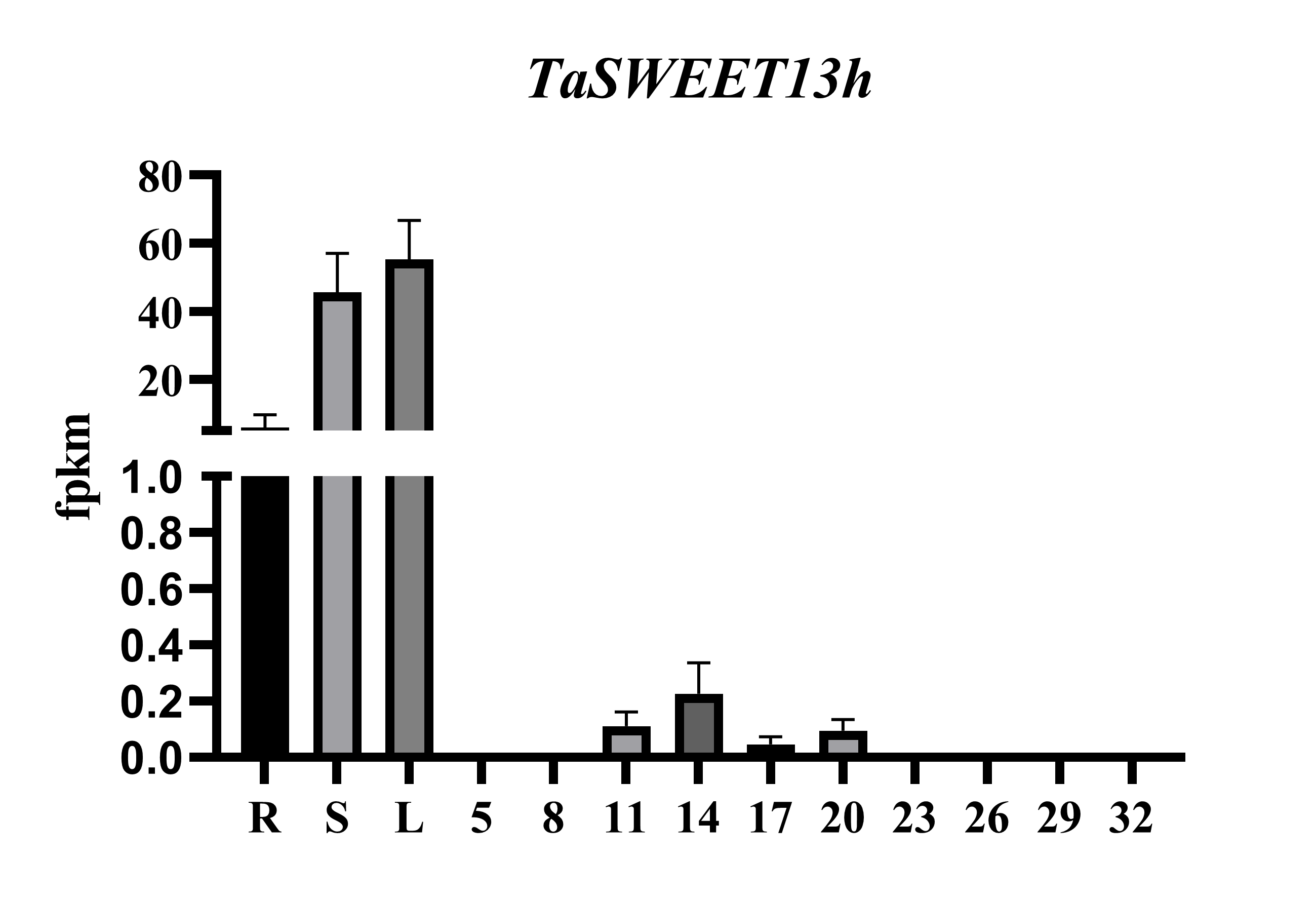


Fig. S2. The expression patterns of the *TaSWEET13h* gene in the grains of Chinese Spring from 5 to 32 DPA (PRJNA545291) and in the roots, stems and leaves at 11 DPA.


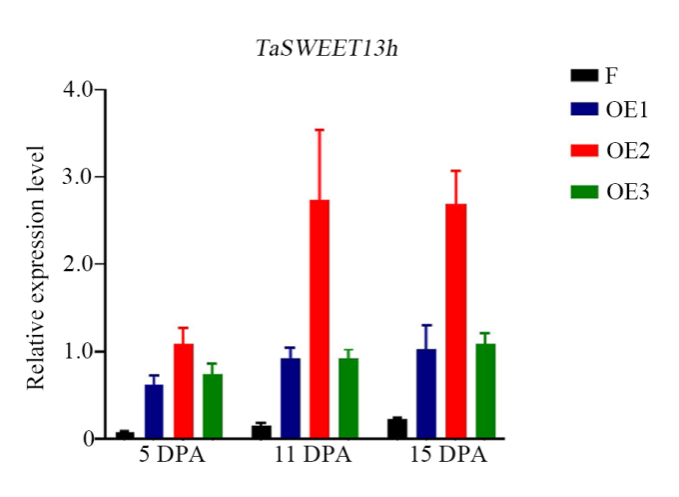


Fig. S3. Expression patterns of *TaSWEET13h* genes in Fielder and *TaDOF6*-overexpression lines (OE1-3) wheat of 5, 11, and 15 DPA grains.


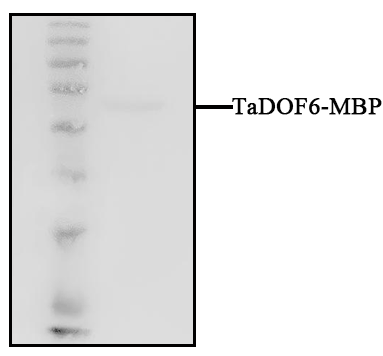


Fig. S4. Detection of the prokaryotic expression protein of TaDOF6 by Western blot.


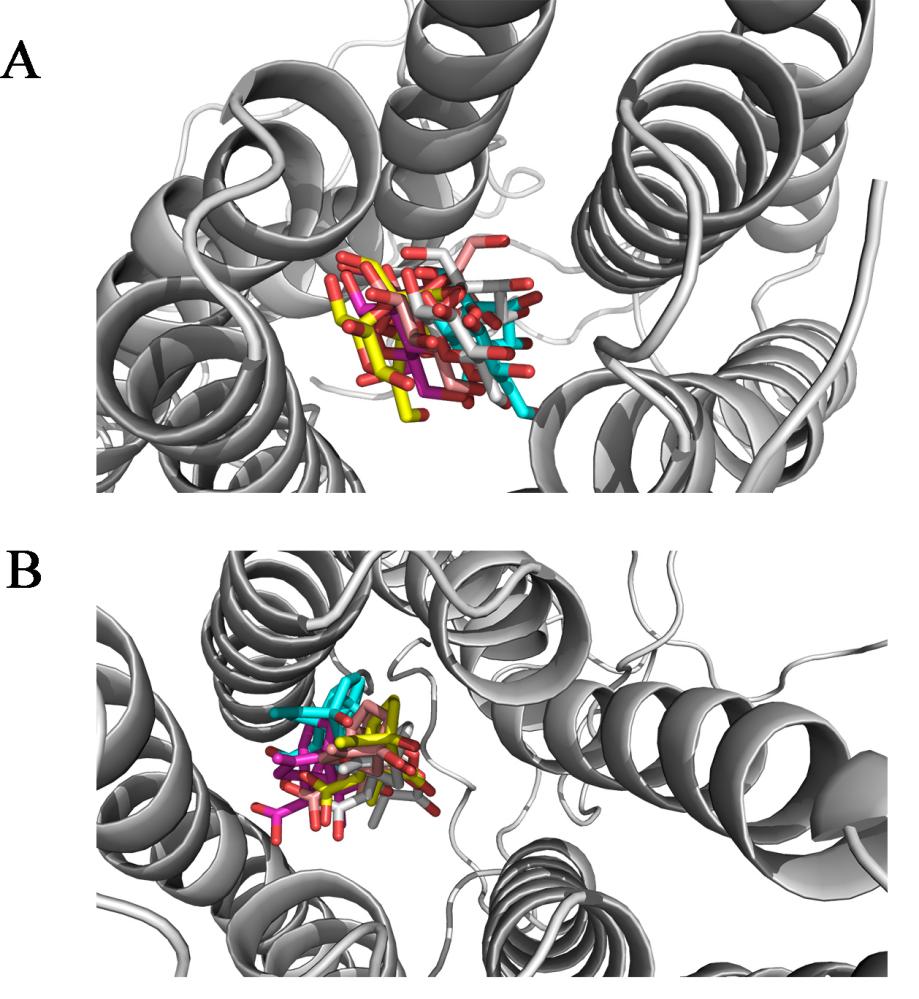


Fig. S5. Superposition of five representative conformations of sucrose (A) and GA_3_ (B) at the active site of TaSWEET13h when the simulation trajectory reached a stable state during the MD simulation. The five conformations are represented by different colors.
